# 

**Published:** 1913-08

**Authors:** 


OFFICIAL ORGAN—State Society Social Hygiene and Eugenics.

VOL. XXIX AUGUST, 1913 No. 2

### The "Red Ba[illegible]k"

### TEXAS MEDICAL JO[illegible]RNAL

Surgeon General, U. S. A.,
Washington, D. C.

ESTABLISHED JULY, 1885

PUBLISHED MONTHLY AT AUSTIN, TEXAS, B[illegible]

F. E. DANIEL, M. D., - - - Editor and Proprietor.

PRINTED BY THE VON BOECKMANN-JONES CO.

Subscription, $1.00 a Year, in Advance. - - - - Single Copies, 10 Cents

Entered at the Postoffice at Austin, Texas, as second class mail matter.

